# Visceral adiposity index and triglyceride/high-density lipoprotein cholesterol ratio in hypogonadism

**DOI:** 10.1590/2359-3997000000236

**Published:** 2017-01-27

**Authors:** Cem Haymana, Alper Sonmez, Aydogan Aydogdu, Serkan Tapan, Yalcin Basaran, Coskun Meric, Kamil Baskoy, Mustafa Dinc, Mahmut Yazici, Abdullah Taslipinar, Cem Barcin, Mahmut Ilker Yilmaz, Erol Bolu, Omer Azal

**Affiliations:** 1 Department of Endocrinology and Metabolism Gulhane School of Medicine Ankara Turkey Department of Endocrinology and Metabolism, Gulhane School of Medicine, Ankara, Turkey; 2 Department of Biochemistry Gulhane School of Medicine Ankara Turkey Department of Biochemistry, Gulhane School of Medicine, Ankara, Turkey; 3 Department of Endocrinology and Metabolism Gulhane School of Medicine Haydarpasa Training Hospital Istanbul Turkey Department of Endocrinology and Metabolism, Gulhane School of Medicine, Haydarpasa Training Hospital, Istanbul, Turkey; 4 Department of Cardiology Gulhane School of Medicine Ankara Turkey Department of Cardiology, Gulhane School of Medicine, Ankara, Turkey; 5 Department of Nephrology Gulhane School of Medicine Ankara Turkey Department of Nephrology, Gulhane School of Medicine, Ankara, Turkey; 6 Department of Endocrinology and Metabolism Memorial Atasehir Hospital Istanbul Turkey Department of Endocrinology and Metabolism, Memorial Atasehir Hospital, Istanbul, Turkey

**Keywords:** Hypogonadism, cardiometabolic risk, visceral adiposity index, triglyceride/high-density lipoprotein cholesterol ratio

## Abstract

**Background:**

Cardiometabolic risk is high in patients with hypogonadism. Visceral adiposity index (VAI) and triglyceride/high-density lipoprotein cholesterol (TG/HDL-C) ratio are the practical markers of atherosclerosis and insulin resistance and independent predictors of cardiaovascular risk. To date, no study has evaluated VAI levels and TG/HDL-C ratio in hypogonadism.

**Subjects and methods:**

A total of 112 patients with congenital hypogonadotrophic hypogonadism (CHH) (mean age, 21.7 ± 2.06 years) and 124 healthy subjects (mean age, 21.5 ± 1.27 years) were enrolled. The demographic parameters, VAI, TG/HDL-C ratio, asymmetric dimethylarginine (ADMA), high-sensitivity C-reactive protein (hs-CRP), and homeostatic model assessment of insulin resistance (HOMA-IR) levels were measured for all participants.

**Results:**

The patients had higher total cholesterol (p = 0.04), waist circumference, triglycerides, insulin, and HOMA-IR levels (p = 0.001 for all) than the healthy subjects. VAI and ADMA and TG/HDL-C levels were also higher in patients than in healthy subjects (p < 0.001 for all). VAI was weakly correlated with ADMA (r = 0.27, p = 0.015), HOMA-IR (r = 0.22, p = 0.006), hs-CRP (r = 0.19, p = 0.04), and total testosterone (r = −0.21, p = 0.009) levels, whereas TG/HDL-C ratio was weakly correlated weakly with ADMA (r = 0.30, p = 0.003), HOMA-IR (r = 0.22, p = 0.006), and total testosterone (r = −0.16, p = 0.03) levels. Neither VAI nor TG/HDL-C ratio determined ADMA, HOMA-IR, and hs-CRP levels.

**Conclusions:**

The results of this study demonstrate that patients with hypogonadism have elevated VAI and TG/HDL-C ratio. These values are significantly correlated with the surrogate markers of endothelial dysfunction, inflammation, and insulin resistance. However, the predictive roles of VAI and TG/HDL-C ratio are not significant. Prospective follow-up studies are warranted to clarify the role of VAI and TG/HDL-C ratio in predicting cardiometabolic risk in patients with hypogonadism.

## INTRODUCTION

Hypogonadism is a syndrome characterized by low testosterone levels and a clinical spectrum of poor libido, energy loss, muscle atrophy, and depression. In addition to fertility disturbance, cardiometabolic risk is increased in patients with hypogonadism ( 1 , 2 ). The prevalence of cardiac and metabolic disorders, such as type 2 diabetes mellitus, hypertension, dyslipidemia, and obesity, are significantly increased in these patients ( 2 , 3 ). However, the mechanism by which cardiometabolic risk increases in patients with hypogonadism remains to be completely elucidated. Inflammation, insulin resistance, and endothelial dysfunction are the major contributors to increased cardiometabolic risk in hypogonadism ( 4 - 8 ). In our previous studies, we observed that even young patients with hypogonadism exhibit endothelial dysfunction, inflammation, and insulin resistance ( 9 - 11 ). However, none of the surrogate markers of endothelial dysfunction, inflammation, and insulin resistance are sufficiently robust to be used as prognostic tools. Thus, a simple, widely available, relatively inexpensive, and generally reproducible marker to predict cardiometabolic risk in patients with hypogonadism is needed.

Visceral adiposity index (VAI) is a mathematical model based on simple anthropometric [body mass index (BMI) and waist circumference (WC)] and metabolic [triglycerides (TG) and high-density lipoprotein cholesterol (HDL-C)] parameters and is considered as a simple surrogate marker of visceral adipose dysfunction ( 12 ). VAI is strongly associated with visceral adiposity measured using magnetic resonance imaging and cardiovascular and cerebrovascular events ( 12 ). Recent data also indicate that hypertriglyceridemia and low HDL-C are key components of the metabolic syndrome and are strongly predictive of coronary artery disease ( 13 , 14 ). The TG/HDL-C ratio is another practical marker of atherosclerosis and insulin resistance and an independent predictor of cardiovascular risk ( 15 - 17 ). The role of TG/HDL-C ratio in predicting cardiometabolic risk has been tested in several metabolic disorders, such as diabetes mellitus, hypertension, chronic kidney disease, and nonalcoholic fatty liver disease ( 18 - 21 ).

To date, no studies have investigated VAI and TG/HDL-C ratio in patients with hypogonadism. Therefore, we designed the present study to answer the following questions: 1. Do VAI and TG/HDL-C ratio differ between patients with hypogonadism and healthy subjects? 2. Are insulin resistance, inflammation, and endothelial dysfunction associated with VAI and TG/HDL-C ratio?

## SUBJECTS AND METHODS

This retrospective analysis was performed by evaluating the database of the Department of Endocrinology and Metabolism, Gulhane Military Medical Academy School of Medicine, Ankara, Turkey. Military service is compulsory for all young men in Turkey, and Gulhane Military Medical Academy School of Medicine is the tertiary medical center for military recruits. Patients with hypogonadism are referred to the Department of Endocrinology and Metabolism for both treatment and follow-up. Some of these patients, generally those living in rural regions, have never received treatment. A total of 273 young patients with hypogonadism were registered between 2007 and 2012. Of these, 64 patients were excluded because of a history of androgen replacement; 27 because of high testosterone levels (200–300 ng/dL); 16 because of liver, kidney, or pulmonary disease; 22 because of a diagnosis other than hypogonadotropic hypogonadism (CHH; i.e., primary hypogonadism, panhypopituitarism, or pituitary adenoma); and 32 because of incomplete data on demographic and metabolic parameters. A total of 112 treatment-naive patients (mean age, 21.7 ± 2.06 years) with congenital CHH were included. The control group included 124 age- and BMI-matched healthy subjects (mean age, 21.5 ± 1.27 years).

A portion of the data for this study population was previously published ( 9 - 11 ). None of the control subjects had a chronic disorder or used any medications, including over-the-counter drugs. All subjects provided informed consent, and the Local Ethical Committee of Gulhane School of Medicine approved the study. This study has been registered with Clinicaltrials.gov (NCT02111434).

Detailed medical histories of all patients were obtained before the study. Height, weight, and WC were measured with the subjects in their underwear. BMI was computed as the ratio of weight to the square of height (kg/m^2^). WC was measured on the line between the iliac crest and the lower costal margin parallel to the ground after subjects exhaled. The pubertal developments of the subjects were assessed according to the Tanner stages. CHH diagnosis was based on a history of failure to undergo spontaneous puberty before 18 years of age and was confirmed with tests demonstrating low serum total testosterone and normal or low gonadotropin levels. Pituitary hormones were evaluated in all patients to exclude panhypopituitarism, and pituitary or hypothalamic mass lesions were excluded by evaluation with magnetic resonance imaging.

### Sample collection and laboratory measurements

For biochemical analyses, all blood samples were collected from the antecubital veins between 08:00 and 09:00 h after overnight fasting. The samples were centrifuged for 15 min at 4,000 × *g* , aliquoted, and immediately frozen at −80°C for analyses. Fasting plasma glucose, total cholesterol, TG, and HDL-C levels were measured by the enzymatic colorimetric method using an Olympus AU-2700 autoanalyzer with reagents from Olympus Diagnostics (GmbH, Hamburg, Germany). Low-density lipoprotein cholesterol level was calculated using Friedewald’s formula ( 22 ). Serum basal insulin, total testosterone, follicle-stimulating hormone, and luteinizing hormone levels were measured by the chemiluminescence method using a UniCel DxI 800 Access Immunoassay System (Miami, FL, USA). Complete blood count was obtained using the Olympus AU-2700 autoanalyzer (GmbH). Insulin sensitivity was calculated by the homeostatic model assessment-insulin resistance (HOMA-IR) using the following formula: HOMA-IR = (insulin × glucose)/405 ( 23 ). Plasma high-sensitivity C-reactive protein (hs-CRP) levels were determined in 58 patients and 69 control subjects. Plasma asymmetric dimethylarginine (ADMA) levels were determined in 59 patients and 33 control subjects using the enzyme-linked immunosorbent assay kit (Immundiagnostik, Bensheim, Germany). The minimum detectable concentration of ADMA was 0.05 µmol/L. The hs-CRP level was determined in serum by the immunoturbidimetric fixed rate method using the Olympus AU-2700 autoanalyzer (GmbH). The intra- and inter-assay coefficients of variation were 5.8% and 3.1%, respectively. The minimum detectable concentration of hs-CRP was 0.07 mg/L. VAI was calculated using the following sex-specific equation ( 13 ):



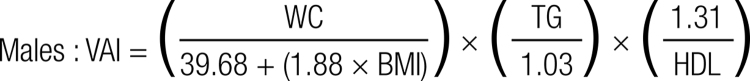



### Statistical analysis

All data were recorded in a computer database and analyzed using the SPSS 18.0 program (SPSS, Inc., Chicago, IL, USA). Results are expressed as means ± standard deviation. The variables were assessed for normality using the Kolmogorov–Smirnov test, and the equality of variance was evaluated using the Levene’s test. Inter-group differences were analyzed using the Student’s *t* -test and Mann–Whitney *U* test as appropriate. The correlations were performed using the Pearson’s or Spearman’s correlation tests. Differences were considered significant at a *p* value of < 0.05.

## RESULTS

**Figure 1:**
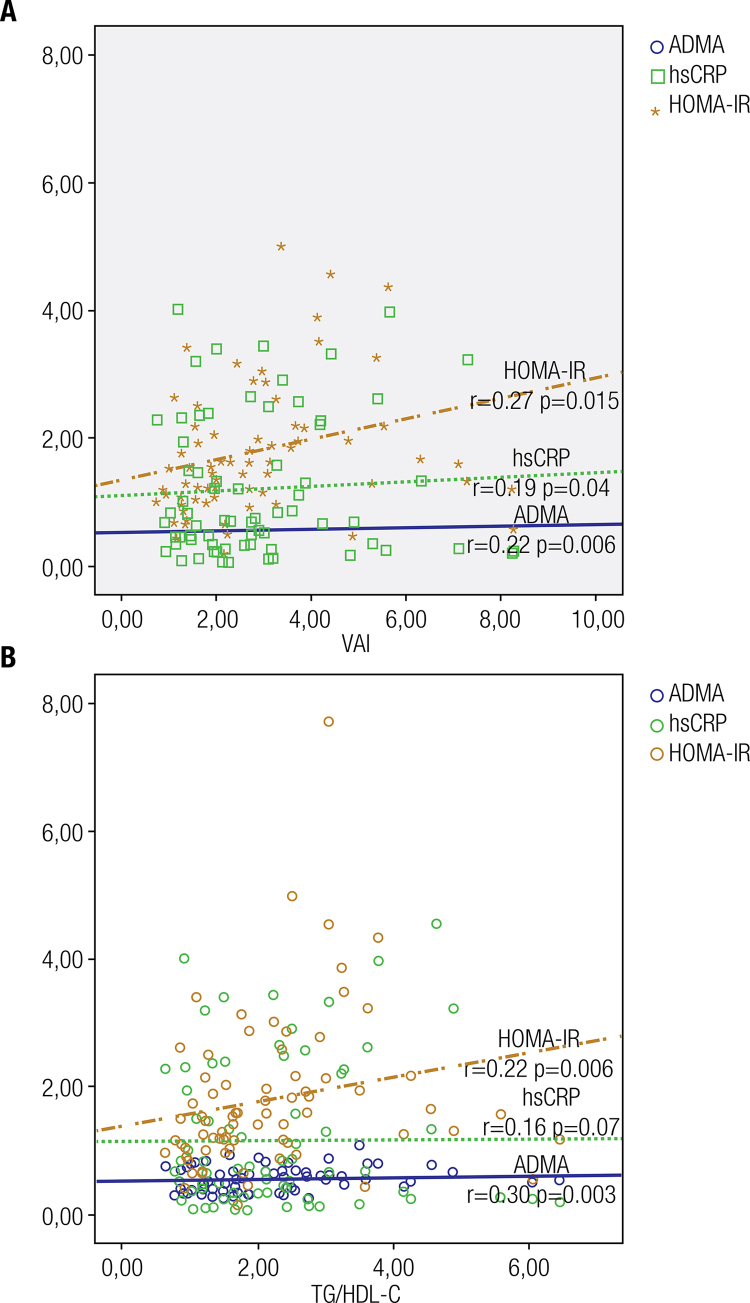
(A) Scatter plot diagram of the correlation between visceral adiposity index (VAI) and asymmetric dimethylarginine (ADMA), high-sensitivity C-reactive protein (hs-CRP), and homeostatic model assessment-insulin resistance (HOMA-IR) levels. (B) Scatter plot diagram of the correlation between triglycerides/high-density lipoprotein cholesterol ratio (TG/HDL-C) and asymmetric dimethylarginine (ADMA), high-sensitivity C-reactive protein (hs-CRP), and homeostatic model assessment-insulin resistance (HOMA-IR) levels.

The demographic and biochemical characteristics of the patients and control subjects are given in Table 1 . Compared with healthy controls, patients had significantly higher total cholesterol levels ( *p* = 0.04); WC, TG, insulin, and HOMA-IR levels ( *p* = 0.001 for all); and ADMA and TG/HDL-C levels and VAI ( *p* < 0.001 for all). VAI were weakly correlated with ADMA (r = 0.27, *p* = 0.015), HOMA-IR (r = 0.22, *p* = 0.006), hs-CRP (r = 0.19, *p* = 0.04; Figure 1A ), and total testosterone (r = −0.21, *p* = 0.009) levels, whereas TG/HDL-C ratio was weakly correlated with ADMA (r = 0.30, *p* = 0.003), HOMA-IR (r = 0.22, *p* = 0.006; Figure 1B ), and total testosterone (r = −0.16, *p* = 0.03) levels. In a stepwise linear regression analysis, neither VAI nor TG/HDL ratio remained in the model to be independent determinants of ADMA or HOMA-IR levels. Total testosterone level was the only significant independent determinant of ADMA level, whereas WC was an independent determinant of HOMA-IR level.

**Table 1:** Demographic and metabolic parameters of patients with congenital hypogonadotropic hypogonadism and healthy control subjects in this study

	Healthy controls (n = 124)*	Patients (n = 112)*	p
Age (year)	21.5 ± 1.27	21.7 ± 2.06	0.31
BMI (kg/m^^2^^)	22.8 ± 2.11	22.17 ± 3.26	0.09
WC (cm)	79.16 ± 6.24	83.61 ± 11.14	**0.001**
SBP (mmHg)	115 ± 10.7	117.2 ± 13.2	0.451
DBP (mmHg)	68.84 ± 5.88	72.07 ± 8.73	0.093
FBG (mg/dL)	84.08 ± 1.0	85.7 ± 7.62	0.16
T. Chol (mg/dL)	151.1 ± 34.1	160.8 ± 26.4	**0.04**
TG (mg/dL)^a^	74.0 (54.7–97.0)	89.0 (63.0–130.5)	**0.001**
HDL-chol (mg/dL)	51.7 ± 15.0	47.1 ± 10.0	**0.007**
LDL-chol (mg/dL)	84.7 ± 30.7	91.6 ± 22.3	0.06
T. testosterone (ng/mL)	550.8 ± 127.7	35.3 ± 42.4	**< 0.001**
Insulin (µU/mL)^a^	6.59 (4.96–8.7)	8.49 (5.65–12.83)	**0.001**
HOMA-IR^a^	1.39 (0.92–1.84)	1.8 (1.18–2.76)	**0.001**
VAI	2.17 ± 1.17	3.22 ± 2.31	**< 0.001**
TG/HDL-C	1.69 ± 0.95	2.35 ± 1.5	**< 0.001**
hs-CRP (mg/L)	0.87 ± 1.21 n = 58	1.18 ± 1.1 n = 69	0.13
ADMA (µmol/L)	0.33 ± 0.6 n = 59	0.66 ± 0.17 n = 33	**< 0.001**

^a^ Mann-Whitney U test. Results are given as means (25% – 75%).

p: Student’s t-test; comparison of parameters between healthy control subjects and patients.

* hs-CRP and ADMA levels are provided separately.

ADMA: asymmetric dimethylarginine; BMI: body mass index; DBP: diastolic blood pressure; FBG: fasting blood glucose; HDL-chol: high-density lipoprotein cholesterol; HOMA-IR: homeostatic model assessment-insulin resistance; hs-CRP: high-sensitivity C-reactive protein; LDL-C: low-density lipoprotein cholesterol; SBP: systolic blood pressure; T. chol: total cholesterol; TG: triglycerides; TG/HDL-C: triglycerides/high-density lipoprotein cholesterol ratio; T. testosterone: total testosterone; VAI: visceral adiposity index; WC: waist circumference.

## DISCUSSION

The results of the present study show that patients with hypogonadism have significantly higher VAI and TG/HDL-C ratio than control subjects. Moreover, VAI and TG/HDL-C ratio are significantly correlated with endothelial dysfunction, insulin resistance, and inflammation (VAI only). However, neither VAI nor TG/HDL-C ratio is applicable as the independent predictor of endothelial dysfunction, inflammation, or insulin resistance in patients with CHH.

Cardiovascular and metabolic disorders, such as obesity, dyslipidemia, hypertension, and type 2 diabetes mellitus, are prevalent in patients with hypogonadism ( 24 - 27 ), but the mechanism by which cardiovascular and metabolic risk increase in hypogonadism is unknown. Inflammation, oxidative stress, insulin resistance, and endothelial dysfunction are the fundamental contributors to increased cardiometabolic risk ( 4 - 8 ). In our previous studies, we reported metabolic derangements and endothelial dysfunction, inflammation, and insulin resistance in young and treatment-naïve patients with hypogonadism ( 9 - 11 ). Patients with hypogonadism have increased fat mass and visceral adiposity, and the latter is associated with an increased risk of developing diabetes, hypertension, dyslipidemia, and atherosclerosis ( 28 , 29 ). We hypothesized that VAI, as an applicable marker for evaluating visceral adipose function, is useful for assessing increased cardiometabolic risk in patients with hypogonadism.

VAI has been recently developed as a novel sex-specific index based on WC, BMI, TG, and HDL-C ( 12 ). VAI is a marker of visceral adipose dysfunction and is strongly associated with cardiovascular events and type 2 diabetes ( 12 , 30 , 31 ). VAI is significantly correlated with inflammation and insulin resistance in patients with type 2 diabetes and cardiovascular disorders ( 12 , 32 ). VAI is also a practical measure for assessing cardiometabolic risk in patients with polycystic ovary syndrome ( 33 ).

TG/HDL-C ratio is another clinical indicator of insulin resistance and has been evaluated as a predictor of diabetes and coronary heart disease ( 34 - 36 ). This ratio may serve as a simpler method for identifying insulin-resistant individuals with increased cardiometabolic risk ( 37 ). TG/HDL-C ratio is also correlated with endothelial dysfunction. In our previous study, we showed that TG/HDL-C ratio is a significant determinant of endothelial dysfunction and a simple predictor of cardiovascular outcomes in patients with chronic kidney disease ( 38 ). Low HDL-C and increased TG levels are also well-established features of patients with hypogonadism ( 9 , 39 , 40 ).

ADMA is an endogenous inhibitor of nitric oxide synthase and a well-known surrogate marker of endothelial dysfunction ( 41 ). Elevated ADMA levels in chronic metabolic diseases, such as type 2 diabetes, hypertension, dyslipidemia, and chronic kidney disease predict cardiovascular morbidity and mortality ( 42 ). We have previously reported elevated ADMA levels in patients with hypogonadism ( 10 , 11 ), which implies increased endothelial dysfunction in this patient group. Most of the markers, such as ADMA, used to define increased cardiometabolic risk in patients with hypogonadism are time-consuming or costly, which precludes their use in routine daily practice. To our knowledge, the present study is the first to measure VAI and TG/HDL-C ratio in patients with hypogonadism.

Our results show that VAI and TG/HDL-C ratio are significantly increased in patients with hypogonadism and are related to markers of inflammation, insulin resistance, and endothelial dysfunction. However, the roles of VAI and TG/HDL-C ratio in predicting endothelial dysfunction, inflammation, and insulin resistance are not sufficiently robust for these parameters to be applicable in clinical practice to predict cardiometabolic risk in patients with hypogonadism. According to the results, total testosterone level and WC are the only independent determinants of endothelial dysfunction and insulin resistance, respectively.

This study has both limitations and advantages. The study population comprising young, treatment-naïve patients with CHH may not be representative of the general population of patients with hypogonadism. Our small sample size may be another limitation. However, because few patients with hypogonadism reach adulthood without receiving treatment, we believe that the number of the patients in our study is adequate because of the unique conditions of the study population. The advantages of the present study are its homogeneous study population and the lack of confounding factors, such as chronic metabolic disorders and concomitant medications.

In conclusion, the present study shows that patients with hypogonadism have elevated VAI and TG/HDL-C ratio, which are significantly correlated with the surrogate markers of endothelial dysfunction, inflammation, and insulin resistance. However, the predictive roles of VAI and TG/HDL-C ratio for endothelial dysfunction, inflammation, and insulin resistance are not significant. Prospective follow-up studies are warranted to clarify the role of VAI and TG/HDL-C ratio in determining cardiometabolic risk in patients with hypogonadism.
